# New model in diabetic mice to evaluate the effects of insulin therapy on biofilm development in wounds

**DOI:** 10.3205/iprs000150

**Published:** 2020-12-23

**Authors:** Jeannine Susanne Schreiter, Christian Beescho, Jagdip Kang, Laura Kursawe, Annette Moter, Judith Kikhney, Stefan Langer, Fredrik Osla, Eric Wellner, Olga Kurow

**Affiliations:** 1 Department of Orthopedics, Trauma and Plastic Surgery, University Hospital Leipzig, Germany; 2 Leipzig Heart Centre, Leipzig, Germany; 3 MoKi Analytics GmbH, Berlin, Germany; 4 Biofilmzentrum, Department for Microbiology, Infectious Diseases and Immunology, Charité – Universitätsmedizin Berlin, Germany; 5 Mölnlycke Health Care GmbH, Goteborg, Sweden

**Keywords:** biofilm, diabetes, insulin, Staphylococcus aureus, wound infection, fluorescence in situ hybridization (FISH), dorsal skinfold chamber

## Abstract

**Objective:** Diabetic patients suffer more frequently from biofilm-associated infections than normoglycemic patients. Well described in the literature is a relationship between elevated blood glucose levels in patients and the occurrence of biofilm-associated wound infections. Nevertheless, the underlying pathophysiological pathways leading to this increased infection vulnerability and its effects on biofilm development still need to be elucidated. We developed in our laboratory a model to allow the investigation of a biofilm-associated wound infection in diabetic mice under controlled insulin treatment.

**Methods:** A dorsal skinfold chamber was used on 16 weeks old BKS.Cg-Dock7^m^ +/+ Lepr^db^/J mice and a wound within the observation field of the dorsal skinfold chamber was created. These wounds were infected with *Staphylococcus aureus* ATCC 49230 (10^6^ cells/mL). Simultaneously, we implanted implants for sustained insulin release into the ventral subcutaneous tissue (N=5 mice). Mice of the control group (N=5) were treated with sham implants.

Serum glucose levels were registered before intervention and daily after the operation. Densitometrical analysis of the wound size was performed at day 0, 3, and 6 after intervention. Mice were sacrificed on day 6 and wound tissue was submitted to fluorescence *in situ* hybridization (FISH) and colony forming unit (CFU) analysis in addition to immunohistochemical staining to observe wound healing.

Experiments were carried out in accordance with the National Institute of Health Guidelines for the Care and Use of Laboratory Animals (protocol number 05/19).

**Results:** The insulin implants were able to reduce blood glucose levels in the mice. Hence, the diabetic mice in the intervention group were normoglycemic after the implantation. The combination with the dorsal skinfold chamber allowed for continuous, in vivo measurements of the infection development. Implantation of the insulin implant and the dorsal skinfold chamber was a tolerable condition for the diabetic mice. We succeeded to realize reproducible biofilm infections in the animals.

**Discussion:** We developed a novel model to assess interactions between blood glucose level and *S. aureus*-induced biofilm-associated wound infections.

The combination of the dorsal skinfold chamber model with a sustained insulin treatment has not been described so far. It allows a broad field of glucose and insulin dependent studies of infection.

## Zusammenfassung

**Einleitung:** Patienten mit Diabetes mellitus leiden häufiger unter Biofilm-assoziierten Infekten als normoglykämische Patienten. Das vermehrte zeitgleiche Auftreten erhöhter Blutzuckerwerte und Biofilm-assoziierter Wundinfekte ist gut beschrieben; der dieser erhöhten Infektvulnerabilität zugrundeliegende Pathomechanismus ist allerdings bisher nicht abschließend erforscht. Um dieses Forschungsfeld künftig tiefergehend zu studieren, haben wir in unserem Labor ein neues Tiermodel zur Untersuchung der Biofilmbildung in diabetischen Mäusen unter einer kontrollierten Insulinbehandlung etabliert.

**Material und Methoden: **16 Wochen alten BKS.Cg-Dock7^m^ +/+ Lepr^db^/J Mäusen wurde in einem Rückenhautkammermodell eine dermale Wunde gesetzt. Diese Wunden wurden mit *Staphylococcus aureus* ATCC 49230 (10^6^ Zellen/mL) infiziert. Zeitgleich wurden den Tieren je nach Versuchsgruppe (je N=5) entweder Insulin verabreichende subkutane Implantate (LinShin Canada Inc., Toronto, Canada) oder wirkstofffreie Implantate appliziert. Die Tiere lebten insgesamt 6 Tage. Täglich wurden das Gewicht, die Temperatur und Blutzuckerwerte der Tiere bestimmt. Nach 6 Tagen wurden Gewebeproben jeweils für Fluoreszenz-*in-situ*-Hybridisierung, CFU und immunhistochemische Färbungen gewonnen. Die Versuche sind entsprechend der Tierversuchsordnung des Landes Sachsen durchgeführt worden (TVV05/19).

**Ergebnisse: **Wir konnten ein neuartiges zuverlässiges Modell zur Untersuchung der Insulintherapie auf die Ausbildung Biofilm-assoziierter Wunden in diabetischen Mäusen etablieren. Die Insulinpumpentherapie konnte eine Reduktion der Blutzuckerwerte erreichen. Die Kombination des Rückenhautkammermodells, welches kontinuierliche In-vivo-Untersuchungen der Infektion erlaubt, mit Insulinimplantaten ist ein für die diabetischen Mäuse gut tolerierbares Modell.

**Diskussion:** Wir haben ein neuartiges Modell zur Untersuchung des Zusammenhangs zwischen Insulin- und Blutzuckerwerten auf die Bildung von Biofilm-assoziierten Wundinfekten am diabetischen Tiermodell entwickelt. Dieses Modell eröffnet ein weites Spektrum an künftigen Forschungsfeldern.

## Introduction

The care of diabetic wounds is a daily challenge in the clinical workload. Diabetic patients suffer more frequently from biofilm-associated infections than normoglycemic patients [1], [2] and there is indeed a known relationship between elevated blood glucose levels in patients and the occurrence of biofilm-associated wound infections [3]. Hyperglycemia hampers the activity of natural killer cells and it may therefore enhance biofilm development [4]. Nevertheless, the exact underlying pathophysiological pathways leading to this increased infection vulnerability and its effects on biofilm development still need to be elucidated. As in vitro models cannot fully simulate the complex in vivo situation, we developed in our laboratory a model to allow the investigation of biofilm-associated wounds in diabetic mice under controlled insulin treatment. 

BKS.Cg-Dock7^m^ +/+ Lepr^db^/J (Charles River, Sulzfeld, Germany) is a mouse strain developing Type II diabetes (NIDDM) and is largely used in hyperglycemia, hyperinsulinemia, and insulin resistance research [5], [6], [7], [8]. Rich et al. [9] showed that within 10 days, animals with a non-treated diabetes suffered from a higher bacterial load than non-diabetic animals in wounds infected with *Staphylococcus aureus*. Hence, within 10 days an infection can be reliably induced in rodents. The model allows for pathophysiological studies. 

The dorsal skinfold chamber is an ideal model for continuous in vivo monitoring and is a well-established technique, which allows for investigations in wounds, angiogenesis, implant, and tumor research [10]. It can be used in mice [10], rats [11] and hamsters [12] and several enhancements of this model enlarging its use have been published so far [13], [14], [15].

We chose the dorsal skinfold model in this study as it prevents skin contraction, which normally occurs in rodents and which would interfere with the observation of the infection process [7]. 

In this study, we combined the dorsal skinfold chamber with a sustained insulin releasing implant in diabetic mice. This novel approach of a rodent model permits the management of blood glucose levels without repeated traumatization of the animals. This model is now ready available to investigate the effects of insulin on biofilm formation and wound healing in a rodent model.

## Materials and methods

### Animals

We used a total of 10 female BKS.Cg-Dock7m +/+ Lepr^db^/J (Charles River, Sulzfeld, Germany). Mice were housed in our own animal facility under standardized environmental conditions, aged in average 16.2 weeks with a mean weight of 50.4 g. The animals were held in individual macrolon cages (Ehret, Emmendingen, Germany) with free access to tab water and normal laboratory chow (ssniff Spezialdiäten GmbH, Soest, Germany). They had a 12 h light and dark cycle; room temperature was 20±2 degrees Celsius.

We chose female mice, as Parks and colleagues showed that estrogens have an insulin sensitizing effect and hence there is a gender specific glycemic response [16]. 

The study was approved by the local governmental animal care committee (protocol number 05/19) and conducted in accordance with the German legislation on protection of animal and the National Institute of Health Guidelines for the Care and Use of Laboratory Animals. The study was performed at the Medical Experimental Centre of the University Hospital Leipzig under S2 laboratory condition.

### Preparation of the animal for the implantation 

After initial weighing and glycemic test, we anaesthetized the mice with an intraperitoneal anesthesia, composed of 500 µg/kg body weight medetomidine (Vetoquinol, Ismaning, Germany), 5 mg/kg body weight midazolam (ratiopharm, Ulm, Germany) and 50 µg/kg body weight fentandon (fentanyl citrate, Dechra, Aulendorf, Germany). We used antisedan (Vetoquinol) 2.5 mg/kg body weight, flumazenil (ratiopharm) 0.5 mg/kg body weight and 1,200 µg/kg body weight naloxone (ratiopharm) subcutaneously to antagonize anesthesia at the end of the operation. During surgery, mice were kept warm on a heating plate at 32°C.

**Figure 1:**
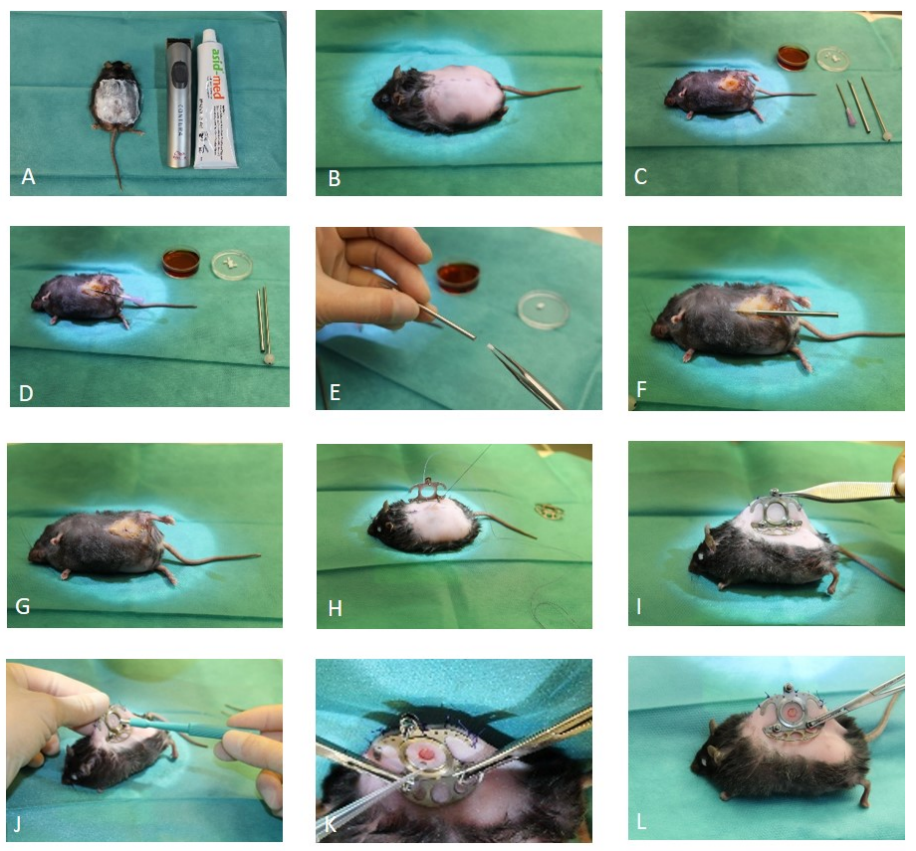
Surgical procedure of the new model, in a female BKS.Cg-Dock7^m^ +/+ Lepr^db^/J mouse. A: Depilation of the mouse with razor and depilation cream. B: Marking of the cranio-caudal line to place the dorsal skinfold chamber. C: Disinfection and preparation of the puncture for the insulin pump placement. D: Perforation of skin using a 12G trocar. E: Placing the insulin implant into the trocar. F: Subcutaneous application of the insulin implant. G: Suture of the puncture wound. H–I: Mounting the dorsal skinfold chamber. J: Wounding. K: Infection of the wound with the *S. aureus* suspension. L: Postoperative appearance.

Afterwards the animals’ dorsal skin was shaved and depilated in an area of 4x8 cm with Asid-med^®^ depilating crème (ASID Bonz GmbH Herrenberg, Germany) (Figure 1A (Fig. 1)), and the cranio-caudal line was marked on the skin (Figure 1B (Fig. 1)).

### Implantation of the insulin implant

We used the sustained insulin release implant from LinShin (LinShin Canada Inc., Toronto, Canada). According to the manufacturer’s recommendation, we implanted two insulin pumps for the first 20 g of mice’s bodyweight. For every additional 5 g bodyweight, an additional pump was implanted. Mean mice body weight was 50.4 g; hence, 8 insulin pumps were implanted on average.

Control animals had Palmitic Acid Micro-Crystal Implants (LinShin Canada Inc.) that did not deliver insulin. 

After disinfection of the ventral side of the animal, we used a 12G trocar (LinShin Canada Inc.) to make an implant pouch (Figure 1C–F (Fig. 1)). After we removed the trocar, we inserted the pump into the space and covered the insertion hole with the mice’s skin. The incision was sutured with Vicryl 4-0 (Ethicon, Norderstedt, Germany) (Figure 1G (Fig. 1)). 

### Preparation of the dorsal skinfold chamber and induction of a standardized wound

We mounted a titanium dorsal skinfold chamber (Hantel GmbH, Heidelberg, Germany) on the mice’s back as previously described by our group [10]. Shortly, two identical titanium frames were sandwiched on the two sides of the dorsal skin (Figure 1H–I (Fig. 1)). On the right side of the animal, in the center of the observation window of the dorsal skinfold chamber, we induced a wound by resecting the dermis and the panniculus carnosus muscle in a diameter 0.4 cm with sterilized micro instruments and a 4 mm punch biopsy (pfm medical, Köln, Germany) (Figure 1 J (Fig. 1)). The wound remained open for the following infection with *S. aureus*. 

### Wound infection with Staphylococcus aureus 

*Staphylococcus aureus* ATCC 49230 was obtained from the Institute for Microbiology of the University Hospital Leipzig. An overnight plate culture was resuspended in 5 mL of casein-peptone-soymeal-peptone broth (Merck KGaA, Darmstadt, Germany) resulting in a suspension with turbidity equivalent to that of a McFarland Standard no. 1 corresponding to 3x10^6^ colony forming units (CFU), which was diluted to a final concentration of approximately 10^6^ CFU/mL. We applied 20 µl of the bacterial suspension with a pipette to the wound in accordance with other *S. aureus* infection animal models [2] (Figure 1K (Fig. 1)). Afterwards, the wound was closed with the cover glass (Figure 1L (Fig. 1)).

### Postoperative analgesia

Mice received Tramadol (Grünenthal, Brunn am Gebirge, Austria) s.c. 25 mg/kg body weight diluted in 0.9% NaCl (6.25 mg/ml) as analgesia. Anesthesia was antagonized at the end of surgery as described before. 

### Intra-vital microscopy and densitometric measurements

Prior to each intra-vital examination, mice were anesthetized by isoflurane (Baxter, Opfikon, Germany) anesthesia, 2% for initializing anesthesia and 0.25% for maintenance of anesthesia. 

Microscopic pictures of the wound in the dorsal skinfold chamber were registered with AxioCam 105 color from Zeiss (Carl Zeiss microscopy, Jena, Germany) at day 0, 3 and 6 after operation. Densitometric measurements of the wound were assessed with Image J [17].

### Blood glucose levels, body weight, and temperature control 

Blood glucose levels were monitored daily using a glucose dipstick (ACCU-Chek Inform II, Roche, Mannheim, Germany). Moreover, body temperature and body weight were assessed daily for both groups. After implantation, the weight of the dorsal chamber (2.338 g) and the weight of the implants (0.014 g x number of implanted devices) was subtracted from the resulting weight.

### Tissue preparation and analysis

Mice were sacrificed by isoflurane overdose, and the wound chambers were aseptically removed.

The wound tissue was divided in one half and two quarters for histology, FISH, and CFU analysis. The quarter to be used for CFU analysis was transferred to 1 ml PBS (pH 7.4) and vortexed for 1 minute to homogenize the biofilm. The bacterial suspension was diluted, 100 µl of each dilution plated on Mueller Hinton agar, and plates were incubated over night at 37°C. Colonies were then counted and final counts were calculated taking the dilution factor into account. For histology, the tissue was placed in zinc-formaldehyde (Sigma Aldrich, Taufkirchen, Germany) and embedded in paraffin, cut in 7 µm sections. Following histochemical dyes can be run according to manufactures’ protocols, depending on the analysis requested.

For FISH analysis, tissue was placed in FISH fixation solution (FISHopt^®^, MoKi Analytics, Berlin, Germany), embedded in methacylate and processed as published previously [18], [19].

### Statistical analyses

This study was designed as a pilot study to establish a new technique to measure the insulin- and glucose effects on *S. aureus* infections and wound healing; hence no power analysis was done. Data graphing was done with GraphPad Prism (GraphPad Prism 8.2.1 for Windows, GraphPad Software, La Jolla California USA, https://www.graphpad.com/).

## Results

### Parameters of the examination groups

Table 1 (Tab. 1) summarizes the data of animals’ weight and age, blood glucose levels and temperature for the two examination groups before operation. No animal died in our experiments. 

### Plasma glucose levels, body weight and temperature

**Figure 2:**
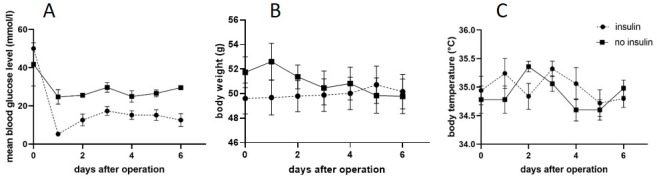
Graph A depicts the blood glucose levels for treated mice (insulin) and the non-treated mice (no insulin). B: Body weight during the experimental procedure (g), C: Body temperature of both experimental groups (°C). All values are mean (SDS), for N=5 in both groups.

Mice’s plasma glucose levels were assessed by dipstick test before anesthesia and daily until day 6. We reached normoglycemic conditions in the treated diabetic mice. Hence, the insulin implant therapy was efficient also under the aggravated situation of the wound infection. Body weight decreased slightly in the animals of the control group, whereas it slightly increased in the intervention group. Body temperature fluctuated around physiological 35°C in both groups. By temperature evaluation and clinical observation of the animals and the wounds, we could exclude a significant systemic infection in our animals (Figure 2 (Fig. 2)).

### Wound characterization and biofilm induction

**Figure 3:**
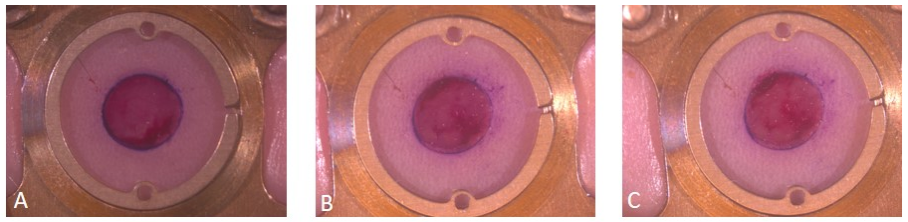
Example of a wound for macroscopic clinical evaluation of the infection and densitometric wound measurements. A: Day 0, after the operation, B: 3 days after the operation; C: 6 days after the operation. We see a locally restricted infection of the wound with pus development. Pictures taken with a stereo microscope and AxioCam 105 color from Zeiss (Carl Zeiss Microscopy).

The here presented model was very well suited for repeated in vivo measurements as for example densitometrical measurements and continuous in vivo angiogenesis research [10]. In this pilot study we verified that we created reproducible wound infections using wound clinical examination and densitometrical analysis (Figure 3 (Fig. 3)). As this is a technical report only, no data are shown in this paper.

### Immunohistochemistry and fluorescence in situ hybridization (FISH)

**Figure 4:**
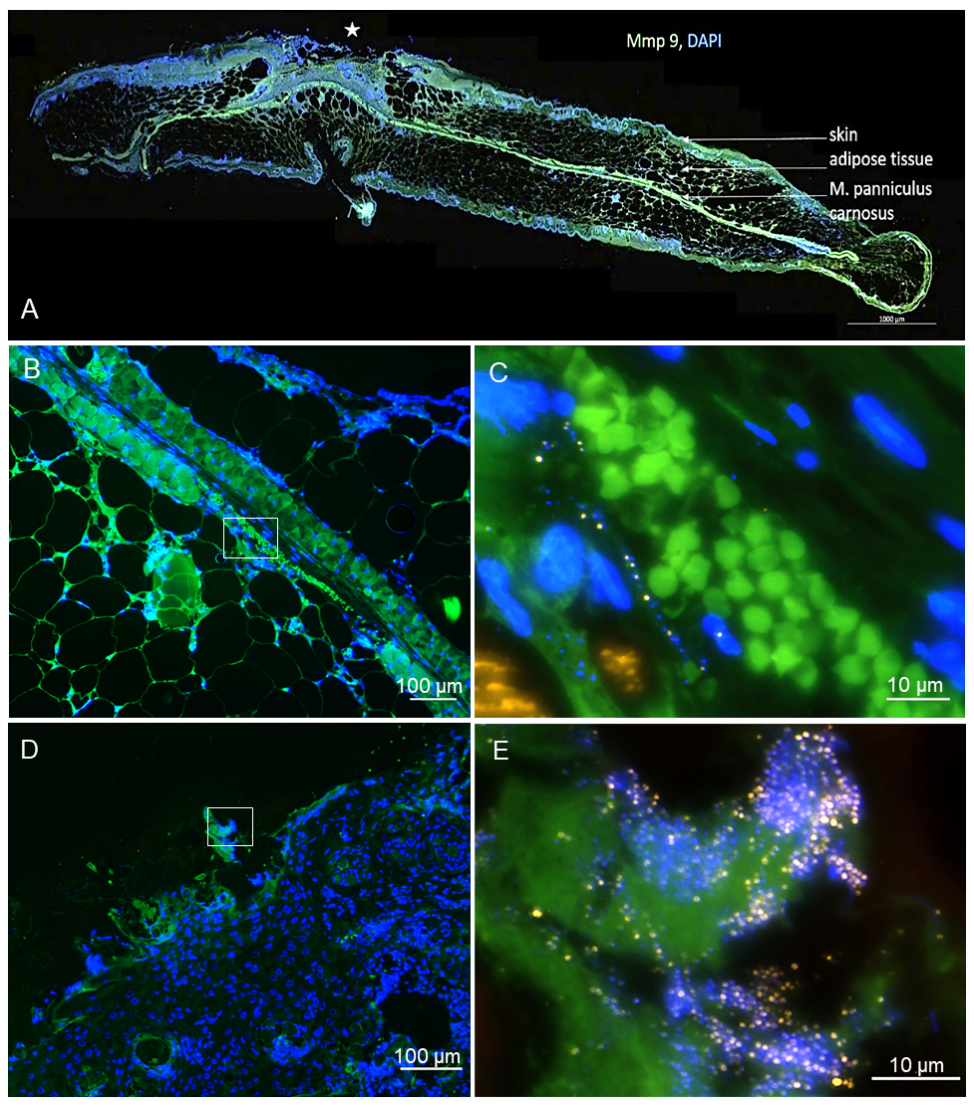
Post mortem wound tissue analysis of a diabetic BKS.Cg-Dock7^m^ +/+ Lepr^db^/J mouse infected with *S. aureus.* A: The immunohistochemical analysis for Mmp9 and DAPI shows a slide of the wound (marked with a star) with the surrounding skin, adipose tissue und panniculus carnosus muscle (arrows). Scale bar: 1,000 µm. Staining by University Leipzig, image courtesy of MoKi Analytics. B–E: FISH analysis of the wound tissue allows visualization of the *S. aureus* distribution. B and C show an example of the outer part of the wound tissue with a biofilm colonizing the surface. In the overview (B), the biofilm is localized on the wound surface (DAPI blue). At higher resolution (C), single FISH-positive bacteria (yellow) are visible, forming a biofilm community. D shows a FISH analysis of deeper layers of the wound tissue with the panniculus carnosus muscle and the subcutaneous fat tissue. In E, bacteria (yellow) are visualized in the wound tissue. Image courtesy of MoKi Analytics.

In addition to in vivo examinations, the model was used for further histological, immunohistochemical, and FISH analysis [20], [21], allowing for host and pathogen analysis in the same model. Inflammation response was studied by macrophage or extracellular matrix dyes, as shown in Figure 4A (Fig. 4). Biofilm could be analyzed and quantified by FISH and compared to CFU measurement results, Figure 4B–E (Fig. 4). FISH confirmed that in a diabetic mouse model, we achieved a reproducible infection with biofilm areas in a wound in a dorsal skinfold chamber.

## Discussion

We successfully established a diabetic mouse model with reliable wound infections in an in vivo dorsal skinfold chamber that can be reproduced with this standardized protocol. Animals showed no signs of systemic inflammation, all animals survived and could have lived for a longer time. 

As the body temperature of the mice did not alter significantly in both groups, we excluded a systemic infection in our animals. We observed a body weight reduction in the control group, while the insulin treated group showed a rather constant body weight during our experiments. This might be an indirect effect of the insulin therapy: The consumption rate in non-treated diabetic mice is usually twice the consumption rate of treated animals. The weight loss in the non-treated animals might be explained therefore by a postoperative weakness, which hampered the food intake and hence lead to lower body weight. In contrast, the lower consumption rate needed in normoglycemic mice was achieved in the intervention group resulting in a stable body weight.

At the beginning of the surgical procedure, blood glucose levels were above the highest measureable levels in the intervention group and almost as high in the control group. In both groups, we saw an initial decrease in blood glucose levels, which is typically occurring after operations. Due to the insulin implants, we reached normoglycemia in the intervention group over the whole experimental period. Though, even without insulin treatment, mice of the control group exhibited lower blood glucose levels after operation than before. This goes in line with the slight body weight loss that we would explain by a postoperative weakness. Although food pellets were dampened with water to facilitate postoperative food intake we suggest to include precise food uptake monitoring for a second study to elucidate this phenomenon of decrease blood glucose levels during the 6 days after the operation. Nonetheless, this does not interfere with the high potential of this model for future studies regarding glycemia and infection analysis. 

The infection with 20 µl of 10^6^ colony forming units (CFU)/mL *S. aureus* in a 4 mm diameter wound allowed for repeated clinical evaluation of the wound regarding open wound measurement, re-epithelialization, and contraction and there was sufficient infection development to endorse pathogen and host response studies. After the in vivo analysis period, the wound tissue could be analyzed regarding histology, CFU counts and FISH. CFU counts indicated a successful monospecies infection with *S. aureus* in all animals, however, the CFU-counts varied substantially. This can be explained by the FISH images, which give literally insights into the exact localization of *S. aureus* within the wounds. Whereas in Figure 4C (Fig. 4), a biofilm at the tissue border is visible, which would lead to high CFU counts, Figure 4E (Fig. 4) shows bacteria infiltrating deep into the wound. Using FISH, it is not only possible to localize the bacteria, but also to estimate their ribosome content, which correlates with the signal intensity in FISH and consequently the activity of the bacteria. Therefore, this model will allow to analyze the influence of insulin and glucose levels on wound healing and inflammation as well as on infiltration, viability and biofilm formation of the bacteria.

## Conclusion

In this present study, we successfully developed a reliable model to assess interactions between different blood glucose levels and *S. aureus* biofilm-associated infection as we succeeded to realize reproducible wound infections in diabetic animals. The model of the dorsal skinfold chamber allows for continuous, in vivo measurements of the infection development. Insulin implants combined with the dorsal skinfold chamber were a tolerable condition for the BKS.Cg-Dock7^m^ +/+ Lepr^db^/J mice. 

The technique described here has not been described so far. It allows for a broad application in glucose dependent studies of infection. Upon variation of the insulin implant dose, the effect of different insulin concentrations on biofilm development can be analyzed in future studies.

This model can be used as well for several blood analyses in the animal followed by different biotechnological studies of the tissue sampled at the end of the study.

## Notes

### Competing interests

The authors declare that they have no competing interests.

### Acknowledgments

The authors greatly thank Ms. Landau, Ms. Köbrig and Ms. Hirrlinger from the University Hospital Leipzig for support at the animal facilities. 

### Funding

This study was financed by Mölnlycke Health Care GmbH, Goteborg, Sweden. 

### Author contributions

Jeannine Susanne Schreiter and Christian Beescho contributed equally.

- Jeannine Susanne Schreiter: writing, original draft preparation, formal analysis, visualization
- Christian Beescho: operated the mice, writing, reviewing
- Jagdip Kang: funding acquisition, conceptualization
- Laura Kursawe, Annette Moter and Judith Kikhney: FISH analysis, visualization
- Stefan Langer, Fredrik Osla and Eric Wellner: conceptualization, project administration
- Olga Kurow: conceptualization, writing the application for animal study, operated the mice, data curation, investigation, supervision

## Figures and Tables

**Table 1:**
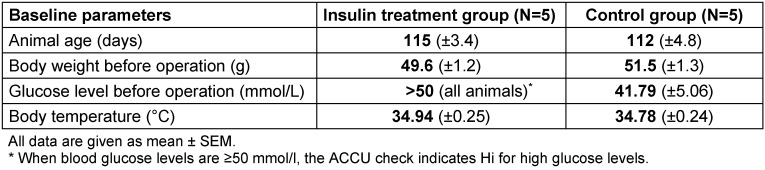
Baseline parameters of the experimental groups (each N=5) before insulin implant and dorsal skinfold chamber implantation
